# Freeze–thaw cycles have minimal effect on the mineralisation of low molecular weight, dissolved organic carbon in Arctic soils

**DOI:** 10.1007/s00300-016-1914-1

**Published:** 2016-03-11

**Authors:** A. Foster, D. L. Jones, E. J. Cooper, P. Roberts

**Affiliations:** 1 School of the Environment, Natural Resources and Geography, Bangor University, Bangor, UK; 2 Department of Arctic and Marine Biology, Faculty of Biosciences, Fisheries and Economics, UiT The Arctic University of Norway, 9037 Tromsø, Norway

**Keywords:** Below-ground respiration, Carbon cycling, Climate change, Freezing temperature, Polar soils

## Abstract

Warmer winters in Arctic regions may melt insulating snow cover and subject soils to more freeze–thaw cycles. The effect of freeze–thaw cycles on the microbial use of low molecular weight, dissolved organic carbon (LMW-DOC) is poorly understood. In this study, soils from the Arctic heath tundra, Arctic meadow tundra and a temperate grassland were frozen to −7.5 °C and thawed once and three times. Subsequently, the mineralisation of 3 LMW-DOC substrates types (sugars, amino acids and peptides) was measured over an 8-day period and compared to controls which had not been frozen. This allowed the comparison of freeze–thaw effects between Arctic and temperate soil and between different substrates. The results showed that freeze–thaw cycles had no significant effect on C mineralisation in the Arctic tundra soils. In contrast, for the same intensity freeze–thaw cycles, a significant effect on C mineralisation was observed for all substrate types in the temperate soil although the response was substrate specific. Peptide and amino acid mineralisation were similarly affected by FT, whilst glucose had a different response. Further work is required to fully understand microbial use of LMW-DOC after freeze–thaw, yet these results suggest that relatively short freeze–thaw cycles have little effect on microbial use of LMW-DOC in Arctic tundra soils after thaw.

## Introduction

A conservative estimate of global soil organic C stocks is about 2200 Pg in the top 1 m (Batjes 1996). There is approximately 472 Pg SOC in the top 1 m of Arctic regions, which is >21 % of the above global estimate, making the Arctic an important store of SOC (Hugelius et al. 2014). However, there is uncertainty as to the fate of this SOC with climate change predicted to increase Arctic temperatures and, potentially, soil temperatures and C degradation. There is also uncertainty as to how increases in air temperature will affect winter soil temperatures. Warmer winter temperatures could increase the occurrence of above 0 °C soil temperatures and melt insulating snow cover, causing freeze–thaw cycles (FTC), where soils freeze and then thaw or vice versa, when air temperatures drop (Henry 2008; Førland et al. 2011). Alternatively, projected increased precipitation could lead to increased snow depth, providing insulation from fluctuating air temperatures and thus warmer, more stable soil temperatures (Førland et al. 2011; Semenchuk et al. 2013). Results from the Canadian Arctic suggest that the occurrence of FTC may increase at high latitudes as global temperatures increase (Henry 2008). FTC have been shown to affect C dynamics in soil with changes to CO_2_ emissions, DOC concentrations and microbial biomass being observed (Schimel and Clein 1996; Larsen et al. 2002; Grogan et al. 2004; Matzner and Borken 2008). However, inconsistencies exist in these observations, and increased mechanistic understanding of FTC effects would be beneficial (Matzner and Borken 2008). It is important to identify what effect FTC may have on C turnover and allocation within Arctic soils to better understand potential feedbacks of climate change (Anisimov et al. 2007).

Most soil organic C (SOC) is not immediately available to heterotrophic microorganisms as it is composed of high MW, insoluble polymers that require extracellular enzymatic cleavage to low molecular weight (LMW) compounds prior to use (van Hees et al. 2005; Farrar et al. 2012). LMW dissolved organic carbon (DOC) is generally considered to contain molecules <1000 Da, and much of it (<650 Da) can be taken up directly into the cell via specific transporters (Payne and Smith 1994). It is produced by the breakdown of larger organic matter, directly from root and microbial exudates and from desorption of molecules precipitated/bound on particle surfaces. LMW-DOC is an important substrate for soil micro-organisms (van Hees et al. 2005). It is comprised of a large variety of compounds including, but not limited to, amino acids, organic acids, amino sugars, mono and polysaccharides, peptides, lipids, sterols and phenolics (Kalbitz et al. 2003; van Hees et al. 2005). Certain compounds within this complex mixture are turned over rapidly by the microbial community and can be major contributors to total soil respiration (Boddy et al. 2007; Fujii et al. 2010).

Freeze–thaw cycles (FTC) have been shown to sometimes, though not always, increase the concentrations of DOC and to affect microbial activity and community structure (Stres et al. 2010; Yu et al. 2011). FTC can produce LMW-DOC by causing damage and fatality to roots and microbes and by breaking up soil aggregates (Tierney et al. 2001; Herrmann and Witter 2002; Henry 2007). Some studies have shown that FTC can induce severe microbial mortality, resulting in large changes in microbial community size and structure (Skogland et al. 1988; Stres et al. 2010; Wilson and Walker 2010). However, these laboratory studies used freezing temperatures and frequencies more extreme than those naturally experienced in soil. Where Arctic or alpine tundra soils which are adapted to lower temperatures were used, less change to the microbial community structure was observed (Männistö et al. 2009; Stres et al. 2010). However, changes in fungal growth relative to bacteria have been shown to occur after FTC, though both increases and decreases have been observed, whilst fungi have been shown to be more active at stable freezing temperatures than bacteria (Feng et al. 2007; McMahon et al. 2009; Haei et al. 2011). Repeated, mild FTC decreased microbial biomass carbon in sub-Arctic tundra (Larsen et al. 2002; Grogan et al. 2004).

How the microbes use the released DOC has not been studied in detail. Once produced, LMW compounds such as amino acids, peptides and glucose are rapidly consumed (within minutes) by the microbial community and the C used for both cell maintenance and growth (Hill et al. 2008, 2012). The subsequent partitioning of the LMW C inside the cell has previously been operationally split into two functional pools: one C pool is used immediately for respiration and the other C pool for biosynthesis before eventually being respired (Boddy et al. 2008; Glanville et al. 2012). A third pool has occasionally been assigned to represent more recalcitrant products of biosynthesis (Farrar et al. 2012). The proportion assigned to each pool depends on the substrate and can provide useful insights into C use efficiency and microbe responses to abiotic and biotic stresses (van Hees et al. 2005). Whether microbial use of LMW-DOC is affected by FTC is less well known, but Lipson and Monson (1998) showed no change in the respiration of glycine or glutamate after a FTC in alpine tundra. Degens et al. (2001) found less variability in the mineralisation of different LMW-DOC compounds in temperate soils after 1FTC to −30 °C, but the variability returned to normal after 4FTC. Further investigation will help illuminate the precise mechanisms that occur during FTC and provide more information on the fate of released DOC.

The aim of this study was to identify how the mineralisation of LMW-DOC is affected by FTC in both an Arctic and temperate soil and to see whether repeated FTC have more or less effect than a single cycle. A number of studies have shown the most damaging effects of FT to be observed within the first couple of cycles (Skogland et al. 1988; Yu et al. 2011) so some difference between 1 and 3FTC may be observed. We tested the hypotheses that FTC will cause a shift in microbial C use efficiency and mineralisation rate, that this change will be compound specific, that FTC will have a greater impact on the mineralisation of LMW-DOC in the temperate soil than the Arctic soil and that 3FTC will have less effect than 1FTC. To achieve this, soils from the Arctic tundra and a temperate grassland were subjected to FTC. Subsequently, ^14^C-labelled LMW-DOC compounds were added and their evolution as ^14^CO_2_ was monitored.

## Methods

### Soils

**Fig. 1:**
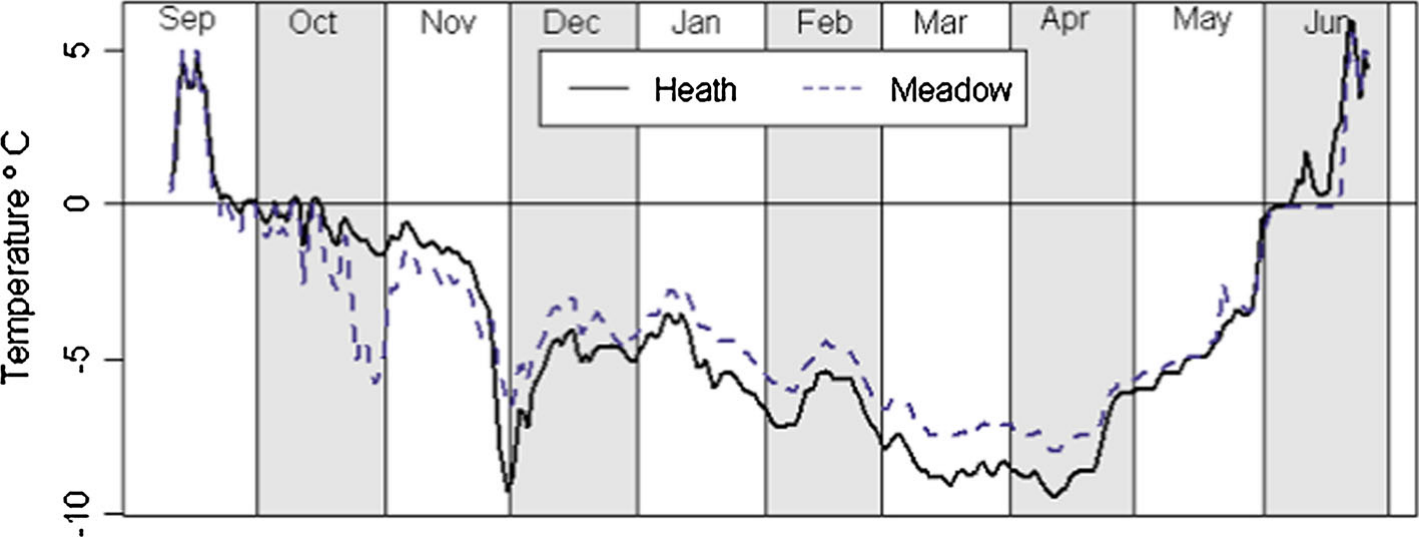
Average daily soil temperatures in the winter of 2012–2013 measured by temperature data loggers at 1 cm depth at the Arctic sites

Individual replicate soil samples (*n* = 4) were collected from three sites. Two contrasting Arctic tundra vegetation sites in Svalbard were chosen: Arctic heath soil (*Dryas octopetala* and bare soil dominated) was sampled from Kolhaugen, Ny Ålesund (78°55.224′N; 11°52.439′E) and Arctic meadow soil (*Salix polaris* and lichen dominated) was sampled from Westbyelva, Ny Ålesund (78°55.4′N; 11°54.4′E). Soil was also sampled from a temperate grassland (*Lolium perenne* and *Trifolium repens* dominated) in Abergwyngregyn, UK (53°14.20′N; 04°01.03′W). The average winter temperature (1981–2009) in Ny-Ålesund, Svalbard, was −12 °C (Førland et al. 2011), whereas the average winter temperature for the same period at the temperate site was 3.4 °C (UK Met Office Statistics). Arctic soils are frozen from October to June and can experience variable snow cover from bare soil to >1 m (Norwegian Meteorological Institute Statistics). The heath site is more exposed than the meadow site and experiences colder soil temperatures (Fig. 1). The temperate soil seldom freezes or experiences snow cover (UK Met Office Statistics). The heath site is approximately 50 m a.s.l, whilst Westbyelva and Abergwyngregyn are 25 m a.s.l.

Both Arctic soils are cryosols, the Arctic heath soil is a sandy mineral soil beneath a very shallow/absent organic layer, whilst the Arctic meadow tundra soil has an organic layer up to 10 cm thick overlying a gravel-rich mineral layer. The temperate soil is a grazed grassland, Eutric Cambisol soil type. Samples were taken randomly over an area of half a hectare. Soils were sampled at a depth of 5 cm over a diameter of 10 cm. The variable thickness of the organic layer at the meadow site meant that the soil sampled at 5 cm was either mineral (meadow mineral) or organic (meadow organic), and replicates (*n* = 4) of both soil types at that depth were collected. The organic layer at the heath site generally did not reach 5 cm so only the mineral soil (heath mineral) was sampled. The overlying vegetation of the precise area sampled was similar for that of the entire site, save in the case of the heath mineral soil which was taken from mostly bare soil areas as the *D. octopetala* cover was patchy. Arctic soils were sampled in July 2012, whilst the temperate soil was sampled in January 2013. After sampling, soils were stored in oxygen permeable bags, stored at 5 °C and transported to Bangor University and subsequently stored at 5 °C.

### Chemical analysis

Soil extractions (1:2.5 v/v soil/deionised water) were performed to assess soil nutrient status. Soil microbial biomass C was measured using the soil fumigation–extraction method of Vance et al. (1997). Briefly 10 g of soil was fumigated with chloroform for 24 h. Then, DOC was extracted from fumigated and non-fumigated soil by shaking in deionised water, and the difference in C content was assumed to represent the microbial biomass C. Fumigated and non-fumigated deionised water extracts were analysed for total organic C (TOC) and total dissolved N (TDN) on a TOC-V-TN analyser (Shimadzu Corp., Kyoto, Japan). Inorganic N (NO_3_^−^ and NH_4_^+^) was measured using the methods outlined by Miranda et al. (2001) and Mulvaney (1996) DON was estimated by subtraction of NO_3_^−^ and NH_4_^+^ from the TDN value. Soil pH was measured in 1:2.5 (v/v soil/water) slurries, whilst moisture content was measured gravimetrically after heating to 105 °C overnight. Total Soil C and N were measured using a Carlo Erba NA 1500 Elemental Analyzer (Thermo Fisher Scientific, Milan, Italy). Soil characteristics are given in Table 1.

**Table 1:** Soil chemical properties used in the freeze–thaw studies

Soil type	pH	Total C (%)	Total N (%)	DOC (mg C/kg)	NO_3_^−^ (mg N/kg)	NH_4_^+^ (mg N/kg)	DON (mg N/kg)	MBC (mg C/kg)
Meadow mineral	6.28 ± 0.12 a	7.92 ± 2.43 b	0.67 ± 0.19 b	34.7 ± 3.3 c	<0.05	2.20 ± 0.07 a	6.04 ± 0.98 b	424 ± 84 b
Meadow organic	6.40 ± 0.16 ab	24.0 ± 2.9 a	1.77 ± 0.06 a	89.5 ± 12.6 b	1.95 ± 0.75 a	2.63 ± 1.52 a	16.9 ± 3.28 a	1989 ± 61 a
Heath mineral	7.39 ± 0.10 b	5.51 ± 2.17 b	0.38 ± 0.18 b	29.1 ± 1.6 c	0.37 ± 0.37 a	<0.05	5.51 ± 0.74 b	368 ± 63 b
Temperate	6.10^ǂ^ ± 0.10 a	3.01^ǂ^ ± 0.16 b	0.23^ǂ^ ± 0.01 b	159.8 ± 13.4 a	2.03 ± 0.85 a	1.90 ± 0.85 a	19.9 ± 3.32 a	610^ǂ^ ± 30 b

Values are indicative of the mean ± SE (*n* = 4). MBC represents microbial biomass carbon. All values are expressed on a dry weight basis where applicable. The letters indicate significant difference between soil types (*p* < 0.05)

^ǂ^Data from Roberts and Jones (2012)

### Freeze–thaw cycles

Treatment consisted of either 1 FTC or repeated (3) FTC and controls (i.e. no FTC) for each FTC treatment. Coarse roots and stones >2 mm were removed by sieving the sampled soils prior to treatment. Fresh soil (2 g) was taken from each replicate for each of the 4 treatments. Soils were frozen to −7.5 °C at a rate of 1 °C h^−1^, and this temperature was maintained for 2 days. Soils were then thawed at a rate of 1 °C h^−1^ to 5 °C. This freezing temperature is representative of winter soil temperatures at the Arctic sites (minimum of −7.5 and −9.4 °C at 1 cm depth for the meadow and heath site, respectively, during winter 2012–2013) and is also seen in other sites in Svalbard (Morgner et al. 2010; Semenchuk et al. 2013). Similarly, rapid changes in soil freezing temperature have also been observed (Semenchuk et al. 2013). In the repeated FTC, this temperature was maintained for 2 days after which the freeze–thaw cycle was repeated a further 2 times. The mineralisation of the LMW C substrates in the soils was tested 12 h after the air temperature had returned to 5 °C after either 1 or 3FTC. Controls soils were maintained at 5 °C.

### DON and DOC mineralisation

The mineralisation rates of LMW DON and DOC compounds were determined for three substrates: the oligopeptide trialanine (231 Da), an amino acid mixture (equimolar mix of L-isomeric alanine, arginine, aspartic acid, glutamic acid, glycine, histidine, isoleucine, leucine, lysine, phenylalanine, proline, serine, threonine, tyrosine and valine, 75–174 Da) and the sugar, glucose (180 Da). For the ^14^C-labelled glucose and amino acid substrates, 200 µl of a 100 µM solution (1.2 kBq ml^−1^) was added to the soil surface. For trialanine, 200 µl of a 10 µM solution (1.4 kBq ml^−1^) was added to the soil. The soils were incubated at 5 °C in sealed 50-ml polypropylene tubes in which an alkali trap (1 ml of 1 M NaOH) was placed to trap the ^14^CO_2_ produced. Traps were exchanged after 0.5, 1, 2, 4, 7, 21, 30, 48, 72 or 96 and 168 h. The ^14^C content of the NaOH traps was determined using a Wallac 1409 scintillation counter (PerkinElmer Corp., Waltham, MA) and Hi-Safe OptiPhase 3 scintillation cocktail (PerkinElmer Corp.). From this, the percentage of the added ^14^C remaining in the soil at each sampling time was calculated by subtracting the % ^14^C emitted from 100.

A double first-order decay model was fitted to the resulting data

1 $$Y = \left( {a_{1} {\text{e}}^{( - k1t)} } \right) + \left( {a_{2} {\text{e}}^{( - k2t)} } \right)$$

where *Y* is the ^14^C remaining in the soil, *a*_1_ and *a*_2_ are the relative sizes of the quickly and more slowly turned over fractions of substrate, respectively, *k*_1_ and *k*_2_ are their associated rate constants and *t* is time. As alanine, trialanine and glucose in the temperate soil have been shown to be taken up in minutes by soil micro-organisms, but only slow, limited sorption observed in sterile soils (Hill et al. 2008, 2012), the quickly turned over substrate fraction can be considered to represent substrate immediately respired by micro-organisms, whilst the more slowly turned over substrate fraction characterises the substrate that is incorporated into biomass which may then be subsequently respired. It should be noted that ^14^C incorporation into the biomass was not physically measured and only the temperate grassland soil has been tested for mineral adsorption. Thus, the capacity of the Arctic soils for the LMW-DOC sorption maybe greater in which case some of the *a*_2_ pool could include substrate bound to soil particles. This model has been shown to be a good fit for the mineralisation over 2 weeks of the LMW DOC substrates used in this study and in a number of environments including temperate grasslands and Arctic tundra (Boddy et al. 2007; Farrell et al. 2011; Glanville et al. 2012). The double first-order exponential decay model was found to generally be a significantly better fit than the single first-order exponential decay model using the extraF.nls function in the FlexParamCurve library of R to perform an F test (Oswald et al. 2012). The parameters of the double first-order exponential decay model were also used to assess carbon use efficiency for the temperate soil as described by Farrell et al. (2011), assuming *a*_2_ represents C used for biosynthesis.

### Statistics

Significant effects of soil type, substrate type, FT treatment and time (the duration of the experiment which was shorter for 1FTC than for 3FTC), plus significant interactions, were assessed using a weighted least-squares full-factorial ANOVA for each parameter derived from the fitted model. Within each soil type, a weighted least-squares full-factorial ANOVA was applied to examine the effect of substrate type, FT treatment and time for that soil. Tukey’s post hoc test was used to identify where significant differences occurred between FT treatment and associated controls within each soil and substrate type. Differences in mineralisation parameters between soil types for the first control were examined using weighted least-squares one-way ANOVA and Tukey’s post hoc test. Only the first controls were used as these would be more representative of initial soil values. Significant differences between soil chemical characteristics were identified using one-way ANOVA and Tukey’s post hoc test. All statistics were performed in SPSS version 20 (SPSS Inc., Chicago, IL). Differences were considered significant where *p* < 0.05.

## Results

### Soil chemical properties

The heath mineral soil had a significantly higher (*p* < 0.001; Table 1) pH than the other soils. No other significant differences were observed in the chemical characteristics of the two Arctic mineral soils (*p* > 0.05; Table 1), whilst the meadow organic soil had a significantly higher soil C (*p* < 0.001; Table 1), N (*p* < 0.001; Table 1), and microbial biomass C (MBC; *p* = 0.004, *p* = 0.008 and *p* < 0.001 for the meadow mineral, heath mineral and temperate soils, respectively; Table 1) than the other soils. The temperate soil had the greatest DOC concentrations (*p* < 0.001 for all soils; Table 1). The DON concentrations were significantly greater in the temperate and meadow organic soils than in the meadow mineral and heath mineral soils (*p* = 0.007 and *p* = 0.006 for the temperate soil, *p* = 0.034 and *p* = 0.026 for the meadow organic soil; Table 1). The pH, soil C and soil N of the temperate soil were not measured in this analysis; values from the same soil type, measured by Roberts and Jones (2012) have been provided for reference.

### Soil mineralisation parameters

**Fig. 2:**
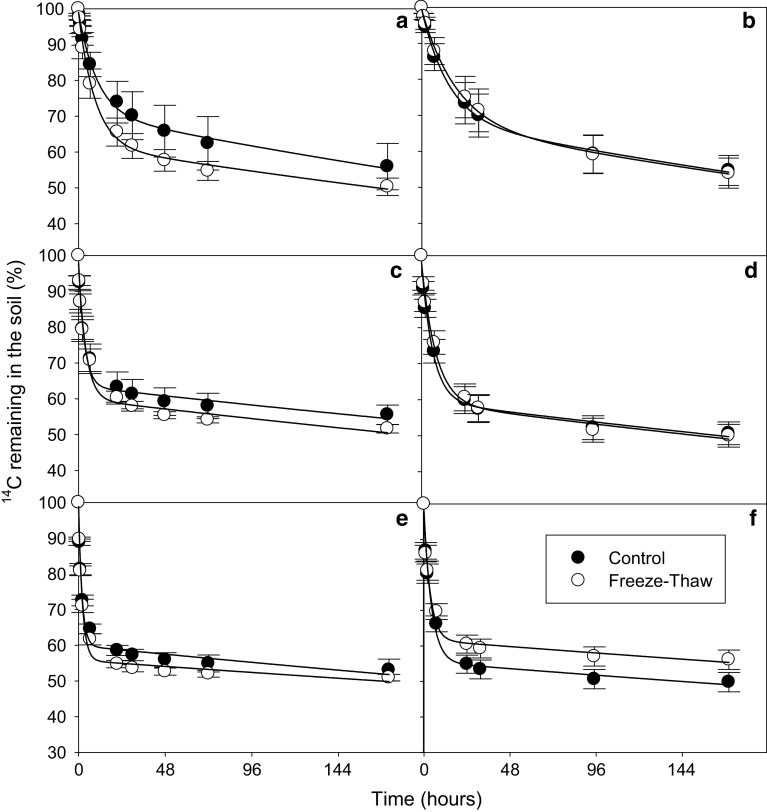
Depletion of ^14^C—added as trialanine—in Arctic tundra soils: Heath mineral soil (**a**, **b**), Meadow mineral soil (**c**, **d**) and Meadow organic soil (**e**, **f**) after they had been subjected to 1 (**a**, **c**, **e**) or 3 (**b**, **d**, **f**) freeze–thaw cycles (−7.5 to +5 °C). The results are fitted with a double first-order exponential decay equation. *Error bars* indicate ± 1SE

**Fig. 3:**
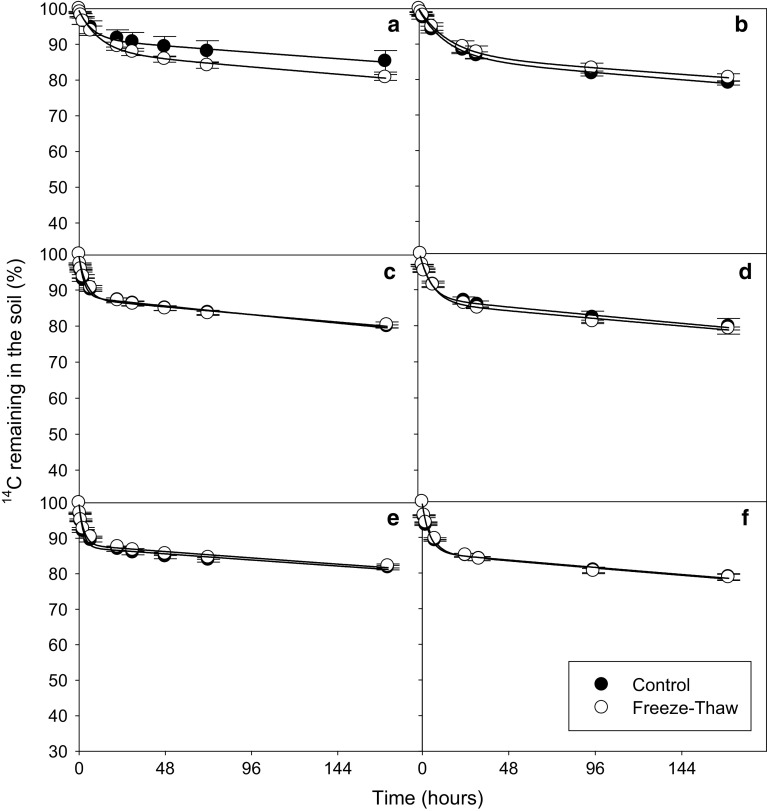
Depletion of ^14^C—added as amino acids—in Arctic tundra soils: Heath mineral soil (**a**, **b**), Meadow mineral soil (**c**, **d**) and Meadow organic soil (**e**, **f**) after they had been subjected to 1 (**a**, **c**, **e**) or 3 (**b**, **d**, **f**) freeze–thaw cycles (−7.5 to +5 °C). The results are fitted with a double first-order exponential decay equation. *Error bars* indicate ± 1SE

**Fig. 4:**
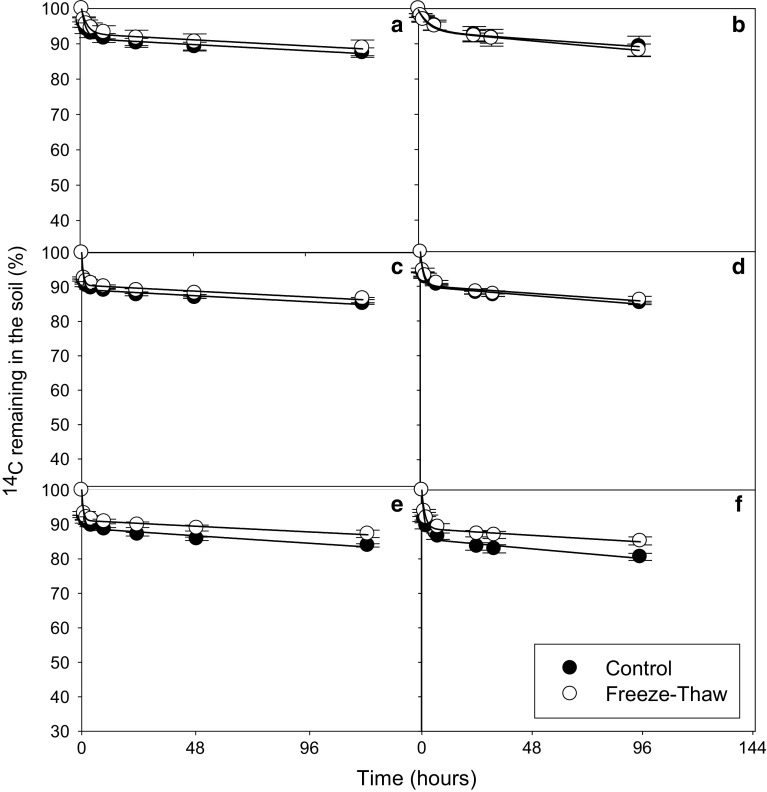
Depletion of ^14^C—added as glucose—in Arctic tundra soils: Heath mineral soil (**a**, **b**), Meadow mineral soil (**c**, **d**) and Meadow organic soil (**e**, **f**) after they had been subjected to 1 (**a**, **c**, **e**) or 3 (**b**, **d**, **f**) freeze–thaw cycles (−7.5 to +5 °C). The results are fitted with a double first-order exponential decay equation. *Error bars* indicate ± 1SE

**Fig. 5:**
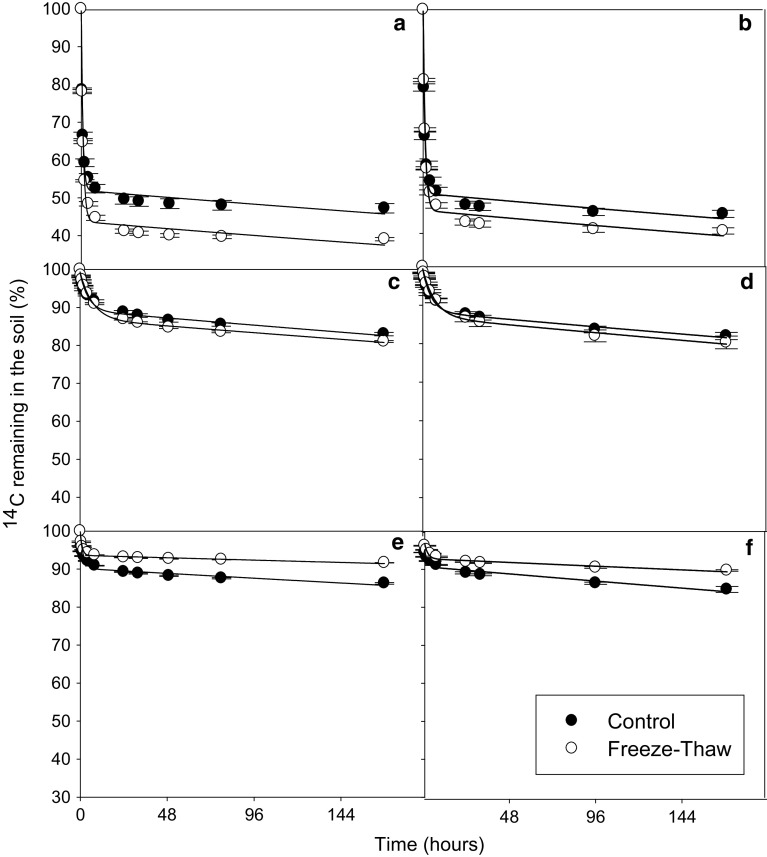
Depletion of ^14^C—added as ^14^C-trialanine (**a**, **b**), ^14^C-amino acids (**c**, **d**) and ^14^C-glucose (**e**, **f**) in temperate grassland soils after they had been subjected to 1 (**a**, **c**, **e**) or 3 (**b**, **d**, **f**) freeze–thaw cycles (−7.5 to +5 °C). The results are fitted with a double first-order exponential decay equation. *Error bars* indicate ± 1SE

**Fig. 6:**
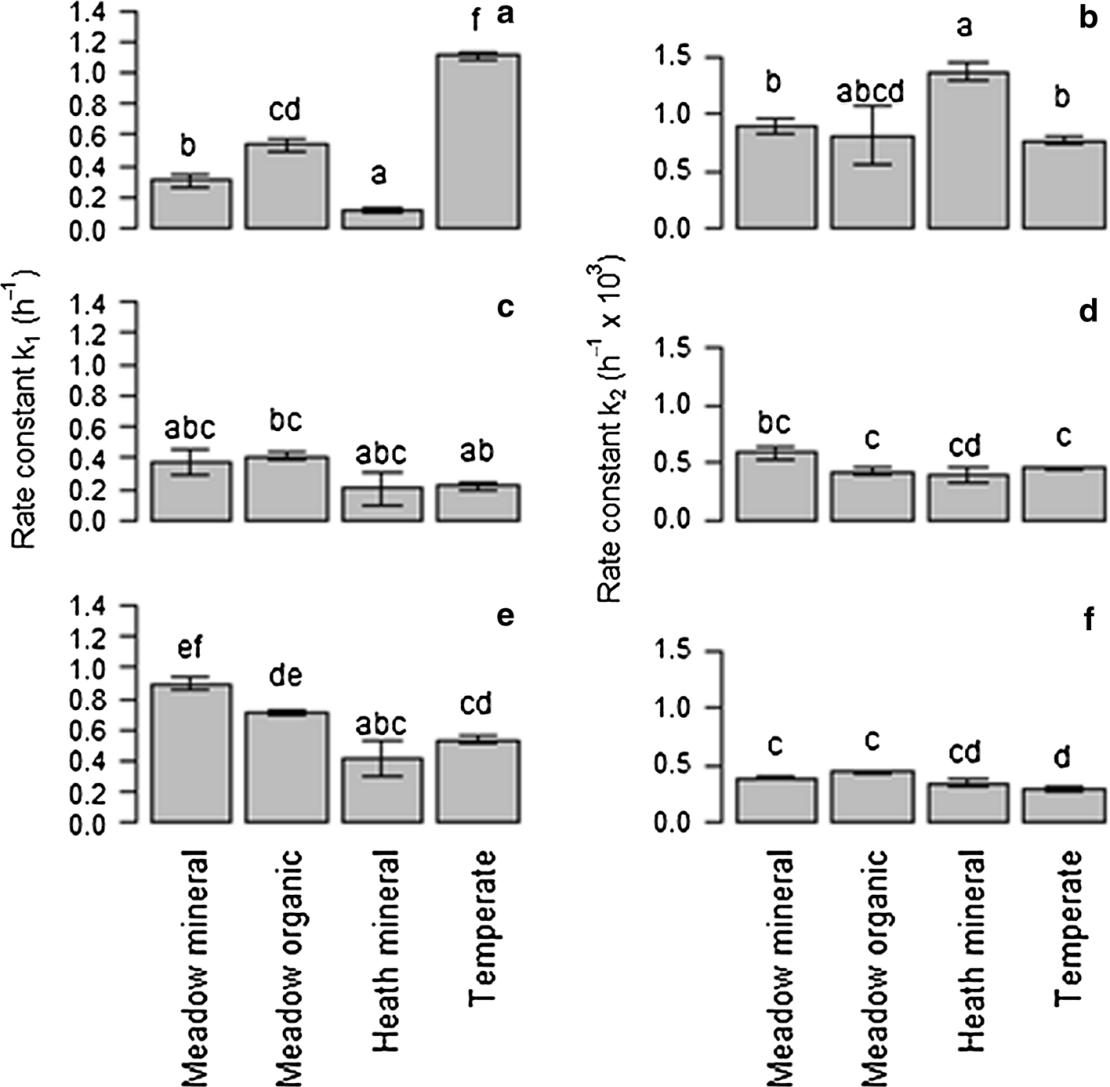
Rate constants (**a**, **c**, **e** parameter *k*_1_; **b**, **d**, **f** parameter *k*_2_) of each soil type after addition of ^14^C-trialanine (**a**, **b**), ^14^C-amino acids (**c**, **d**) and ^14^C-glucose (**e**, **f**) in the first control sample set. *Letters* reveal significant difference within each parameter comparing across soil and substrate (*p* < 0.05). *Error bars* indicate ± 1SE

**Fig. 7:**
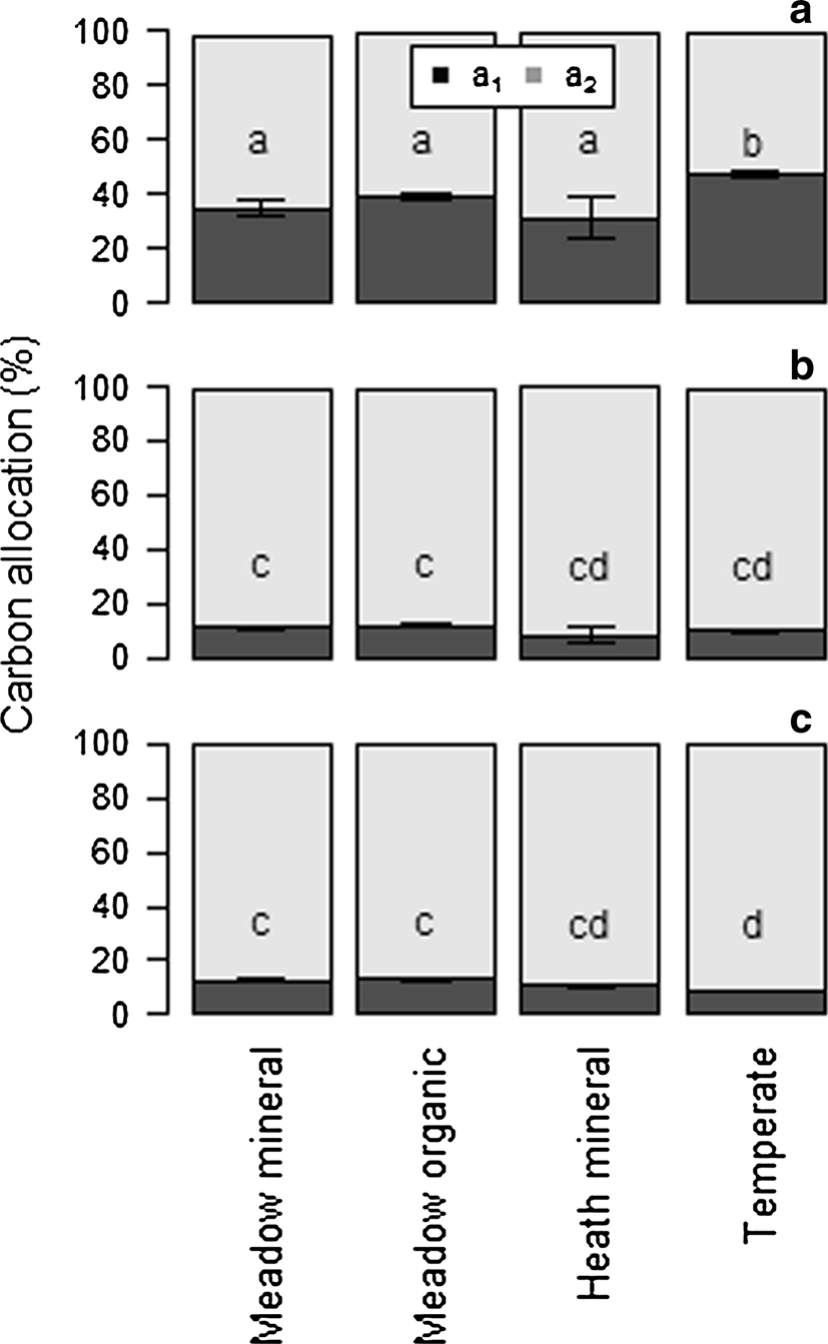
Carbon allocation (parameters *a*_1_ and *a*_2_) in each soil and substrate after addition of ^14^C-trialanine (**a**), ^14^C-amino acids (**b**) and ^14^C-glucose (**c**) in the first control sample set. *Letters* reveal significant difference across soil and substrate (*p* < 0.05). *Error bars* indicate ± 1SE

In all soils and for all substrates, a biphasic pattern of mineralisation was observed. A double-exponential decay model was found to fit well to this experimental mineralisation data (*r* ^2^ = 0.971 to >0.999) (Figs. 2, 3, 4, 5). A significant effect of both soil and substrate was observed for all mineralisation parameters (*p* < 0.001 for all). There were significant differences in the modelled mineralisation parameters for the first cycle controls between soil types (Figs. 6, 7; Tables 2, 3, 4). The first rate constant (*k*_1_) describing the rate of trialanine turnover was significantly different between the soil types, with the temperate soil being the quickest (*p* < 0.001), followed by the meadow organic soil (*p* = 0.012) and then the meadow mineral soil (*p* = 0.003), with the heath soil having the slowest turnover (*p* Table 2 *k*_1_ control 1; Fig. 6). In contrast, no significant difference in turnover rate was observed between soil types for the amino acid substrate (Table 3 *k*_1_ control 1; Fig. 6). The turnover of glucose showed some difference between soil types. Glucose turnover in the temperate soil was slower than in the meadow soils (*p* < 0.001 for both), whilst the heath soil had a significantly slower glucose turnover than the meadow mineral soil (*p* = 0.027; Table 4 *k*_1_ control 1; Fig. 6). The rate constant for the second, slower C pool (*k*_2_) was not significantly different in any soil type for amino acids, but for trialanine the heath mineral soil had a significantly greater *k*_2_ than the meadow mineral soil and the temperate soil (*p* = 0.004 and *p* < 0.001, respectively; Table 2 *k*_2_ control 1; Fig. 6). For glucose, the turnover of the second C pool in the temperate soil was slower than in both meadow soils (*p* = 0.007 and *p* < 0.001 for the mineral and organic soil, respectively; Table 4 *k*_2_ control 1; Fig. 6).

**Table 2:** Modelled double first-order kinetic parameters describing the mineralisation of ^14^C-labelled trialanine in Arctic and temperate soils subjected to 1 or 3 successive freeze–thaw (FT) cycles (−7.5 to +5 °C) and their respective non-frozen controls

Trialanine					
Soil	Parameter	Control	FT	3 Control	3FT
Meadow mineral	*a*_1_ (%)	34.6 ± 3.7	37.8 ± 1.4	38.8 ± 3.4	39.9 ± 0.9
	*k*_1_ (h^−1^)	0.31 ± 0.05	0.24 ± 0.08	0.16 ± 0.03	0.13 ± 0.03
	*a*_2_ (%)	63.5 ± 4.0	60.1 ± 1.4	59.3 ± 3.7	58.2 ± 0.9
	*k*_2_ (× 10^−3^ h^−1^)	0.88 ± 0.06	1.02 ± 0.19	1.03 ± 0.07	0.98 ± 0.14
Meadow organic	*a*_1_ (%)	39.4 ± 1.2	41.8 ± 1.5	43.2 ± 1.5	38.8 ± 4.3
	*k*_1_ (h^−1^)	0.54 ± 0.04!	0.47 ± 0.03	0.24 ± 0.02!	0.34 ± 0.07
	*a*_2_ (%)	59.3 ± 1.1	57.0 ± 1.4	54.9 ± 2.7	59.7 ± 4.5
	*k*_2_ (×10^−3^ h^−1^)	0.81 ± 0.26	0.68 ± 0.05	0.78 ± 0.12	0.66 ± 0.05
Heath mineral	*a*_1_ (%)	31.4 ± 7.4	29.6 ± 10.1	34.2 ± 5.8	34.1 ± 3.9
	*k*_1_ (h^−1^)	0.12 ± 0.01	0.22 ± 0.15	0.08 ± 0.01	0.06 ± 0.01
	*a*_2_ (%)	68.1 ± 7.7	69.6 ± 10.4	65.1 ± 5.9	65.3 ± 4.0
	*k*_2_ (×10^−3^ h^−1^)	1.37 ± 0.09	1.00 ± 0.29	1.33 ± 0.11	1.34 ± 0.16
Temperate	*a*_1_ (%)	47.43 ± 1.2*	55.43 ± 0.7*	48.87 ± 1.2	53.16 ± 0.7
	*k*_1_ (h^−1^)	1.1 ± 0.03*	0.9 ± 0.03*	1.1 ± 0.06	0.8 ± 0.02
	*a*_2_ (%)	52.01 ± 1.2*!	43.73 ± 0.7*	50.73 ± 1.1!	46.17 ± 0.8
	*k*_2_ (×10^−3^ h^−1^)	0.8 ± 0.04	0.9 ± 0.05	0.8 ± 0.01	1.0 ± 0.05

* A statistical difference between freeze–thaw treatment and the control

^!^ Statistical difference over time (i.e. between the first and third control or 1FT and 3FT), (*p* < 0.05). Values represent mean ± SE (*n* = 4)

**Table 3:** Modelled double first-order kinetic parameters that describe ^14^C-labelled amino acid mineralisation in Arctic and temperate soils subjected to 1 and 3 freeze–thaw (FT) cycles of −7.5 to +5 °C

Amino acids					
Soil	Parameter	Control	FT	3 Control	3FT
Meadow mineral	*a*_1_ (%)	11.2 ± 0.8	11.5 ± 0.7	10.8 ± 0.8	12.9 ± 0.2
	*k*_1_ (h^−1^)	0.37 ± 0.08	0.24 ± 0.04	0.15 ± 0.03	0.14 ± 0.02
	*a*_2_ (%)	88.1 ± 0.8	87.5 ± 0.7	88.4 ± 0.8	86.2 ± 0.2
	*k*_2_ (×10^−3^ h^−1^)	0.59 ± 0.06	0.53 ± 0.07	0.47 ± 0.15	0.53 ± 0.03
Meadow organic	*a*_1_ (%)	12.0 ± 0.7	11.2 ± 0.3!	13.3 ± 0.5	13.7 ± 0.5!
	*k*_1_ (h^−1^)	0.41 ± 0.03!	0.37 ± 0.05	0.22 ± 0.02!	0.19 ± 0.02
	*a*_2_ (%)	87.4 ± 0.7	88.1 ± 0.4!	85.9 ± 0.6	85.5 ± 0.5!
	*k*_2_ (×10^−3^ h^−1^)	0.43 ± 0.03	0.44 ± 0.02	0.54 ± 0.04	0.52 ± 0.03
Heath mineral	*a*_1_ (%)	8.6 ± 2.5	12.1 ± 0.5	13.5 ± 0.3	12.9 ± 1.7
	*k*_1_ (h^−1^)	0.20 ± 0.11	0.10 ± 0.04	0.11 ± 0.04	0.06 ± 0.01
	*a*_2_ (%)	90.9 ± 2.6	87.4 ± 0.4	86.0 ± 0.4	86.9 ± 1.6
	*k*_2_ (×10^−3^ h^−1^)	0.39 ± 0.06	0.47 ± 0.04	0.55 ± 0.02	0.45 ± 0.04
Temperate	*a*_1_ (%)	9.9 ± 0.5*	12.4 ± 0.3*	11.0 ± 0.6	12.5 ± 1.0
	*k*_1_ (h^−1^)	0.22 ± 0.02	0.14 ± 0.00	0.21 ± 0.01*	0.15 ± 0.01*
	*a*_2_ (%)	89.2 ± 0.5*	86.9 ± 0.3*	88.3 ± 0.6	86.9 ± 1.1
	*k*_2_ (×10^−3^ h^−1^)	0.46 ± 0.01	0.44 ± 0.01	0.47 ± 0.03	0.50 ± 0.05

The parameters *a*_1_ and *a*_2_ are the proportion of substrate mineralised rapidly or slowly, whilst *k*_1_ and *k*_2_ are their respective rate constants

* A statistical difference between freeze–thaw treatment and the control (not frozen)

^!^ Statistical difference over time (i.e. between the first and third control or 1FT and 3FT), (*p* < 0.05). Values represent mean ± SE (*n* = 4)

**Table 4:** Modelled double first-order kinetic parameters that describe the mineralisation of ^14^C-labelled glucose in Arctic and temperate soils subjected to 1 and 3 freeze–thaw (FT) cycles (−7.5 to +5 °C)

Glucose					
Soil	Parameter	1 Control	1 FT	3 Control	3 FT
Meadow mineral	*a*_1_ (%)	12.6 ± 0.3!	11.3 ± 0.6	10.4 ± 0.3!	10.1 ± 0.6
	*k*_1_ (h^−1^)	0.89 ± 0.04!	0.89 ± 0.06!	0.66 ± 0.05!	0.56 ± 0.06!
	*a*_2_ (%)	87.2 ± 0.3!	88.1 ± 0.6	89.3 ± 0.3!	89.6 ± 0.6
	*k*_2_ (×10^−3^ h^−1^)	0.39 ± 0.01	0.38 ± 0.03	0.41 ± 0.04	0.34 ± 0.04
Meadow organic	*a*_1_ (%)	13.1 ± 0.5	10.7 ± 0.7	14.0 ± 1.0	11.1 ± 0.9
	*k*_1_ (h^−1^)	0.72 ± 0.01	0.89 ± 0.05	0.67 ± 0.03	0.68 ± 0.05
	*a*_2_ (%)	86.6 ± 0.5	89.1 ± 0.7	85.8 ± 1.1	88.8 ± 0.9
	*k*_2_ (×10^−3^ h^−1^)	0.44 ± 0.02	0.36 ± 0.04	0.72 ± 0.01	0.46 ± 0.06
Heath mineral	*a*_1_ (%)	10.7 ± 0.9	9.1 ± 2.1	6.0 ± 1.9	5.9 ± 1.2
	*k*_1_ (h^−1^)	0.41 ± 0.11	0.34 ± 0.07	0.19 ± 0.02	0.24 ± 0.04
	*a*_2_ (%)	89.0 ± 1.0	90.3 ± 2.2	93.5 ± 2.0	93.8 ± 1.3
	*k*_2_ (×10^−3^ h^−1^)	0.35 ± 0.03	0.34 ± 0.03!	0.51 ± 0.1	0.66 ± 0.08!
Temperate	*a*_1_ (%)	8.9 ± 0.1*	6.2 ± 0.1*	8.6 ± 0.3*	6.8 ± 0.2*
	*k*_1_ (h^−1^)	0.54 ± 0.03*	0.93 ± 0.08*!	0.70 ± 0.06	0.55 ± 0.03!
	*a*_2_ (%)	90.2 ± 0.1*	93.6 ± 0.1*	90.7 ± 0.4*	92.8 ± 0.2*
	*k*_2_ (×10^−3^ h^−1^)	0.30 ± 0.01*	0.14 ± <0.01*!	0.44 ± 0.07	0.22 ± 0.01!

The parameters *a*_1_ and *a*_2_ are the proportion of substrate mineralised rapidly or slowly, whilst *k*_1_ and *k*_2_ are their respective rate constants

* A statistical difference between freeze–thaw treatment and the control (not frozen)

^!^ Statistical difference over time (i.e. between the first and third control or 1FT and 3FT), (*p* < 0.05). Values represent mean ± SE (*n* = 4)

The partitioning of glucose- and trialanine-derived C into either slowly respired C (parameter *a*_2_—indicative of allocation to biosynthesis or mineral sorption) or immediate respiration (parameter *a*_1_) was different between the soil types. The temperate soil showed a lower proportion of glucose-derived C allocated to rapid respiration than soil from the meadow site (*p* < 0.001 for both; Table 4 *a*_1_ control 1; Fig. 7). However, proportionally more trialanine was initially used for respiration in the temperate soil than in the Arctic soils (*p* = 0.014, *p* = 0.006 and *p* < 0.001, for the meadow mineral, meadow organic and heath soils, respectively; Table 2 *a*_1_ control 1; Fig. 7). The allocation of amino acid-derived C was not significantly different between soil types (*p* > 0.05; Table 3 *a*_1_ control 1; Fig. 7).

### Influence of freeze–thaw cycles on substrate mineralisation

Freeze–thaw treatment only had a significant effect on mineralisation parameter *k*_1_ when comparing all soil and substrates (*p* = 0.043), but there were significant interactions between soil and FT treatment for all parameters except *k*_2_ (*p* = 0.001, 0.034, 0.013 for parameters *a*_1_, *k*_1_ and *a*_2_). The FT treatment had little effect on the mineralisation parameters in the Arctic soils (Figs. 2, 3, 4, 5; Tables 2, 3, 4, significant difference indicated by *). The Arctic soils showed no significant difference between the FTC treatment and its control for any substrate after either 1 or 3 FT cycle. However, it should be mentioned that there was relatively large variability between replicate results, particularly for the heath mineral soil, which might have obscured any treatment effects.

The temperate soil showed a greater response to FT treatment than the Arctic soils (Figs. 2, 3, 4, 5) with FT having a significant effect for *a*_1_, *k*_1_ and *a*_2_ (*p* < 0.001, *p* = 0.013 and *p* < 0.001, respectively). There was also significant interaction between FT treatment and substrate for all mineralisation parameters (*p* < 0.001). For all three substrates, the rate constant *k*_1_ was significantly affected by FTC. It decreased for both trialanine and amino acids significantly or almost significantly after both 1 and 3 FTC (by 0.20 ± 0.05 (*p* = 0.005) and 0.08 ± 0.02 h^−1^ (*p* = 0.103) after 1FTC and by 0.25 ± 0.06 (*p* = 0.055) and 0.06 ± 0.01 h^−1^ (*p* = 0.001) after 3FTC, for trialanine and amino acids, respectively). The rate constant *k*_1_ for glucose increased after the first cycle, but appears to decrease after 3FTC although there was also an increase in the controls *k*_1_ over time. For glucose, the amount of substrate C allocated to pools *a*_1_ and *a*_2_ changed after both 1 and 3FTC (*a*_1_ decreased by 2.76 ± 0.03 and 1.85 ± 0.20 % (*p* < 0.001 for both) after 1 and 3FTC, respectively). For amino acids and trialanine, the amount of C allocated to pools *a*_1_ and *a*_2_ were only significantly affected by 1FTC (trialanine—*a*_1_ increased by 8.00 ± 1.34 % (*p* < 0.001), alanine—*a*_1_ increased by 2.51 ± 0.53 % (*p* = 0.023). A greater proportion of the trialanine and amino acid-derived C was allocated to initial respiration (pool *a*_1_), whilst the opposite effect was observed in glucose. For glucose, the rate constant for the second C pool (*k*_2_) was significantly lower after 1FTC (by 0.16 ± 0.01 × 10^−3^ h^−1^); for the other substrates, *k*_2_ was not significantly affected (*p* > 0.05). There was significant interaction in the temperate soil between substrate and treatment and time for *a*_1_, *k*_1_ and *a*_2_ (*p* = 0.037, <0.001 and =0.013, respectively). Less of the observed differences between the 3FTC treatment and its associated control were significant than between 1FTC and its control. However, the only significant differences observed between the 1FTC treatment and the 3FTC treatment were in *k*_1_ and *k*_2_ for glucose, and the change observed was not as large as the insignificant change between their respective controls.

## Discussion

### Freeze–thaw effects on C mineralisation in different soils

Although the changes in C mineralisation we observed here in response to FTC were very small, in agreement with our initial hypothesis, temperate soils seem to be more susceptible to FTC than Arctic soils. Freezing can reduce substrate and water supply to microbes, causing starvation and desiccation, whilst subsequent thawing can induce a rapid change in the osmotic gradient, leading to cell lysis (Wilson and Walker 2010). Previous studies have suggested that FTC are most damaging when the microbial community is not adapted to them and where the freezing temperature is more extreme than they would naturally experience (Stres et al. 2010; Wilson and Walker 2010). The freezing temperatures used in this study are similar to minimum winter soil temperatures experienced at the Arctic sites in 2012–2013, although they are colder than soil temperatures experienced in 2013–2014 (−3 °C at the meadow site and −4 °C at the heath site), which was a milder year with deeper snow cover [max—131 cm, 45 cm at Ny-Ålesund in 2013–2014 and 2012–2013, respectively (Norwegian Meteorological Institute Statistics)]. The temperate soil rarely experiences freezing temperatures or snow cover. However, as some characteristics of the temperate soil were different to Arctic soils (Table 1), namely DON, DOC, MBC and potentially others that have not been measured, there could be an alternative explanation for the greater effect shown by temperate soils. For example, different nutrient availabilities in the temperate soils could support a more active microbial community, which is damaged more by FTC, as suggested by Schimel and Clein (1996).

The mineralisation parameters could be affected by substrate being adsorbed to soil particles. Guggenberger and Kaiser (2003) argued that in soil, mineral grains are already largely covered in organic materials and microbes, which would greatly reduce the capacity of minerals to adsorb fresh OM. This is supported by studies showing rapid microbial uptake of LMW-DOC compounds (Hill et al. 2008, 2012). However, Guggenberger and Kaiser (2003) also argued that younger, low organic C soil had more capacity to take up DOC. Therefore, some mineral adsorption could have occurred in the Arctic soils, which may have dampened the effect of FTC.

There is some tentative evidence that suggests substrate mineralisation was less effected after 3FTC than after 1FTC as an interaction was observed between substrate, FT treatment and time. This is similar to some past studies where the adverse effects of FT have been shown to decrease with repeated cycles (Skogland et al. 1988; Larsen et al. 2002; Goldberg et al. 2008; Yu et al. 2011), although Schimel and Clein (1996) suggested that up to 3 repeated FT might still have a damaging effect, and other studies show maximum effect after 2FTC (Morley et al. 1983; Koponen et al. 2006; Yu et al. 2011). Whilst adverse effects decrease, significant recovery of microbial biomass during repeated FTC has not been observed (Morley et al. 1983; Skogland et al. 1988). There was no evidence that 3FTC has more of an effect than 1FTC in any of the soils.

### Substrate-specific freeze–thaw effects in the temperate soil

The changes in mineralisation parameters for amino acids and trialanine were consistent with each other. The changes for glucose differed to the other compounds and were less consistent. Where the kinetic parameter *k*_1_ was affected, it decreased for amino acids and trialanine. For glucose, there was no trend in the change in *k*_1_. For amino acids and trialanine, *a*_1_ increased due to FT and *a*_2_ decreased, whilst the opposite result was observed for glucose. This, combined with the *k*_1_ results, suggests that more trialanine and amino acids were used for mineralisation, but they were used more slowly. The *k*_2_ parameter was only affected by FT for glucose in the temperate soil, where it significantly decreased.

It is unlikely that any change in the mineralisation parameters was caused by a change in microbial community structure, size or activity as these have been shown to have little effect on LMW DOC mineralisation (Jones 1999; Strickland et al. 2010; Rousk et al. 2011). Jones et al. (2005, 2009) found the use of amino acids and peptides to be widespread amongst soil micro-organisms so it is unlikely that a change in community would affect usage.

Strickland et al. (2010) found that P availability was important in predicting glucose mineralisation, in addition to land use and plant cover. FTC could potentially liberate organic phosphate compounds by cell lysis or soil-bound phosphates by the break-up of soil aggregates (Freppaz et al. 2007). This would be likely to increase the mineralisation rate as was observed for glucose after 1FT, but that is not seen here for amino acids or trialanine. It is possible that the disintegration of soil aggregates could create fresh surfaces for phosphates to bind with, which could reduce the mineralisation rate (Özgül et al. 2012). Fresh surfaces could also have bound the added substrate, reducing the ^14^C available for mineralisation. This could lead to a decrease in *a*_1_ and an increase in *a*_2_, but this was only observed for glucose.

Increases in the concentration of LMW DOC components due to FT could increase turnover time as there could be competition for uptake transporters (Jones and Hodge 1999). Farrell et al. (2014) found a decrease in trialanine uptake when very high glucose concentrations (9 mM) were added, which could occur close to areas of cellular lysis after FTC. However, decreased uptake should cause an increase in *a*_2_, which was not observed for trialanine or amino acids. Uptake rate from soil has been considered to be equal to *k*_1_ multiplied by the concentration of substrate in solution (Farrell et al. 2011). Thus, an increase in soil substrate concentration caused by FTC could lead to an increase in microbial uptake rate despite a decrease in *k*_1_.

If one assumes that *a*_2_ represents substrate used for biosynthesis whilst *a*_1_ is the proportion of substrate used for catabolism, an assessment of C use efficiency can be made. For the temperate soil, where the changes in mineralisation parameters were observed due to FTC, this is a fair assumption as rapid microbial uptake, but little mineral sorption of LMW-DOC compounds has been observed (Hill et al. 2008, 2012). For amino acids and trialanine, a decrease in C use efficiency appears to have occurred as more substrate was used for respiration than growth. This could be consistent with a lag phase after FT. Such a lag phase was shown in the results of Drotz et al. (2010). The opposite result was observed for glucose making a lag phase less likely. However, glucose can be used by, or to make metabolites for, most bacterial metabolic systems (Cartledge et al. 1992). It is possible that opposing results could be caused by differences in internal substrate use. Amino acids and trialanine could also be used as an N source should the C/N ration be high. This could result in the C being used for respiration after the deamination (Apostel et al. 2013).

The *k*_2_ parameter was only affected by FT for glucose in the temperate soil. In the instances, where this parameter was significantly affected it consistently decreased. The value of *k*_2_ is dependent on the turnover of the microbial community, and this is influenced by a number of factors including grazing by organisms, such as protozoa, temperature, infection by viruses, heterolysis and substrate availability (Alexander 1981). It also depends on the biochemical pathway of the added substrate, for example the allocation of the substrate C into cell wall structures, that tend to be slower to degrade compared to cytoplasm material, such as metabolites (Malik et al. 2013). The *k*_2_ value could also be affected by desorption of mineral bound C, but, as mentioned above, it is unlikely that much sorption occurs in the temperate soil. The decrease in *k*_2_ suggests a slower turnover time for biosynthesised glucose. If the microbial turnover had decreased, it would be expected that the *k*_2_ values of amino acids and trialanine would also decrease. This is not the case. It seems more likely that the glucose was used to produce less readily degradable material after FT. Further research is required to identify whether this is the case.

### C substrate turnover in soil

C cycling in Arctic soils is typically dominated by the input and breakdown of plant residues (Bird et al. 2002). These residues are dominated by cellulose/hemicellulose and protein whose extracellular cleavage produces simple sugars, peptides and amino acids (Kögel-Knabner 2002). The results describing the mineralisation of these compounds in our Arctic soil were similar to those presented for tundra heath by Boddy et al. (2008) and clearly showed a rapid turnover of this C within the soil solution. The amino acid half-life $$\left( t_{{{\raise0.7ex\hbox{$1$} \!\mathord{\left/ {\vphantom {1 2}}\right.\kern-0pt} \!\lower0.7ex\hbox{$2$}}}} = \frac{{{ \ln }\,2}}{{k_{1} }} \right)$$ for the temperate grassland calculated in our study (3.2 h) was similar to that measured by Jones et al. (2005) for the agricultural soil (2.9 h), but greater than that measured for a similar soil from the same location (0.9 h) (Jones et al. 2005). This could be due to the soils being collected at different times (Glanville et al. 2012) or the much shorter mineralisation measurement period and warmer incubation temperature used by Jones et al. (2005). Farrar et al. (2012) measured longer turnover times for glucose in a similar temperate grassland soil than was found here. Few studies have looked at peptide mineralisation despite their importance in soil C and N cycling. Farrell et al. (2011) found a slightly slower turnover of trialanine (*k*_1_ = 0.77 h^−1^ (Farrell et al. 2011) compared to 1.11 h^−1^) in a similar temperate grassland soil to that used in our study. This is despite the fact that they used a higher incubation temperature (10 °C compared to 5 °C), which has been shown to increase *k*_1_ in temperate soils (Jones 1999). The turnover of trialanine in the Arctic soils appears to be slower than any of the soils analysed by Farrell et al. (2011, 2013). This could be due to the lower experimental temperature and less available nutrients in the Arctic soils (Strickland et al. 2010; Farrell et al. 2013). Overall, we conclude our results describing substrate mineralisation in the control (non-FTC) soils are broadly similar to other studies providing confidence in this indicator to evaluate an FTC effect.

### Implications and experimental caveats

Whilst the temperatures used in this study were representative of air temperature fluctuations typically experienced at the Arctic sites (Norwegian Meteorological Institute Statistics), it is unlikely that soils below the surface experience such extreme and rapid temperature fluctuations. They are insulated by surrounding soil and vegetation (Henry 2007). Furthermore, freezing and thawing soils require more energy than changing its temperature so a sustained period above freezing is required to fully thaw the soil even at the surface. FTC carried out over a narrow temperature range cause less damage than more intense freezing temperatures (Hentschel et al. 2008; Elliot and Henry 2009), so if a warmer freezing temperature had been used, we would have expected a smaller response. As little significant effect was observed at what was likely a relatively extreme FTC for these Arctic soils it appears that an increase in rapid air temperature fluctuations around 0 °C in the Arctic will have limited effect on the use of LMW DOC by soil microbes after thaw. It is possible that a longer freezing period than used in this experiment might have more effect on the microbial use of LMW DOC, since longer freezing periods have been shown to be more damaging to the microbial community than shorter freezing periods (Haei et al. 2011).

These results suggest rapid recovery of microbial utilisation of LMW-DOC after freezing in Arctic soils. This would likely lead to the rapid use of the compounds tested in this study if they were produced during FTC, with microbes being able to capitalise on any flux of LMW-DOC. Therefore, it is unlikely that FTC or repeated FTC would cause increased leaching of these particular compounds. This might explain why some studies have found little effect of FTC on the aromaticity of DOC in leachate if they were thawed for days rather than hours (Hentschel et al. 2008; Vestgarden and Austnes 2009). Rapid recovery of microbes might mean that they could out-compete plants for any LMW organic N produced after FTC. Arctic plants can recover in 12 h from mild freezing and grow in frozen soil, but how rapidly they recover from more intense FTC needs to be determined to show whether this is the case (Billings et al. 1977). Whether microbial utilisation of larger compounds and other microbial processes recovers as quickly as their use of LMW-DOC could be further investigated.

## Conclusions

The aims of this study were to identify whether FTC caused any change in the dynamics of LMW DOC mineralisation. The results described above indicate that short FTC induces a small change in LMW DOC mineralisation on soil from a temperate environment. However, FTC appeared to have little effect on the mineralisation of LMW DOC in Arctic soils. Therefore, it seems unlikely that an increase in Arctic FTC will directly affect the microbial utilisation of LMW DOC after FTC.

The changes in modelled mineralisation parameters due to FT, observed in the temperate soil, varied with the LMW DOC compound. The changes observed for amino acids and trialanine were consistent with each other, showing longer turnover times and more C immediately allocated to respiration after FT. The response for glucose to FT was less clear, and the change in C allocation was opposite to that of amino acids and trialanine, with a lower proportion of the C allocated to respiration due to FT. The reason for this difference requires further investigation.
